# Suggestions for attenuation of renal ischemia reperfusion injury based on mechanisms involved in epithelial cells damages

**Published:** 2015-01-01

**Authors:** Majid Tavafi

**Affiliations:** ^1^Department of Anatomy, Faculty of Medicine, Lorestan University of Medical sciences, Khoram Abad, Iran

**Keywords:** Ischemia, Reperfusion, Kidney, Proximal tubules

**Implication for health policy/practice/research/medical education:**

Major renal injuries follow ischemia reperfusion take place in two steps: one, ischemia (hypoxia) that induce ATP depletion and leads to epithelial tubular cells or endothelial cells injuries; and second, reperfusion that leads to oxidative stress, nitrosative stress and inflammation.

Renal I/R (ischemia reperfusion) occurs in kidney surgeries such as partial nephrectomy or transplantation because of renal vessels ligation during the procedures. Ischemia (tissue hypoxia) leads to renal damages especially proximal convoluted tubules (PCT) injuries (1-3). Serious damages also occur during and following reperfusion. Mechanisms that I/R damage proximal tubules cells are briefly explained below.

Ischemia decreases ATP production and finally leads to tissue ATP depletion. ATP depletion leads to Rho GTPase inactivation that makes activation of ADF (Actin depolymerizing factor) or cofilin in the apical brush border of proximal tubules (4-6). Activated cofilin (ADF) rapidly depolymerizes apical actin cytoskeleton and redistribution. Deterioration of microvillar structure leads to formation of membrane blebs, which may be either internalized or shed into the tubular lumen. Brush border membrane components that are released into the lumen give to cast formation and tubular occlusion (7).

ATP depletion also dissociates the actin-stabilizing proteins such as tropomyosin and ezrin (8), permitting the activated cofilin to bind and then depolymerize actin, which finally leads to microvillar breakdown. Activation of cofilin also can induce apoptosis in PCT cells by inducing release of cytochrome C from mitochondria to cytoplasm (9).

PCT cell death occurs from at least two cell death mechanisms, necrosis and apoptosis. Activation of cofilin after ATP depletion also can induce apoptosis by activating intrinsic pathway (9) and also extrinsic pathway. Do not forget inhibition of apoptosis acts as double blade sword because, inhibition of apoptosis will promote survival of injured or mutation-bearing cells in other organ systems (10). Remember that apoptosis is the powerful normal mechanism for removal of genomic damaged cells.

Challenges for the future clinical use of apoptosis inhibition in acute kidney injury (AKI) include determining the best timing of therapy, optimizing the specificity of inhibitor, minimizing the extra renal adverse effects, and tubule-specific targeting of the apoptosis modulatory maneuvers (10). In my opinion inhibition of apoptosis inducer such as cofilin in this issue is safer than inhibition of apoptosis pathways.

Interruption of the apical cytoskeleton by ATP depletion also results in loss of tight junctions and adherents junctions between tubular cells and leads to tubular cells disconnection (11).

Ischemia leads to disruption of at least two proteins, Na, K-ATPase and integrins. Deterioration of basolateral Na, K-ATPase in PCT is cause of increasing in excretion of sodium in tubular lumen (10). Integrins are in basal region of PCT and mediate cell connection to basal lamina. Ischemia leads to relocalization of integrins to the apical membrane, and then makes detachment of PCT cells from the basement membrane. Preischemic intravenous administration of anti-activated β1 integrin resulted in preservation of renal histopathology and function, maintenance of cell binding to basal lamina (12).

Some PCT cells maybe remain viable after I/R and undergo recovery. These cells show the appearance of dedifferentiated epithelial cells, and then the cells up regulate genes that encode some growth factors and undergo proliferation and undergo re-differentiation until the normal epithelium is restored (13).

Cofilin also degrades actin cytoskeleton and junctional complexes in endothelial cells in experimental AKI (14) and leads to endothelial cell swelling, blebbing, death, and detachment of viable cells (15). Sites of endothelial denudation may be prone to prolonged vasoconstriction, and cessation and even reversal of blood flow in peritubular capillaries during reperfusion (10). Furthermore, ischemia induces apoptosis of endothelial cells (16).

After ischemia and restoration of blood flow, production of reactive oxygen species (ROS), such as superoxide, hydroxyl, H2O2 and activation of leukocytes and endothelial cells contribute to reperfusion injury (17).

Decrease of innate antioxidant enzymes, increase of intracellular sodium and calcium, stimulation of NO synthetize, endonucleases and phospholipases also occur during reperfusion (18). Nitric oxide changes to peroxynitrite and then leads to injury by inducing inflammation.

Ischemic proximal tubule cells also generate mediators such as proinflammatory cytokines (e.g., TNF-α, IL-6, IL-1β, and TGF-β) and chemotactic cytokines such as monocyte chemo attractant protein-1 (MCP-1) and IL-8 (19).

There are many studies that administration of antioxidant agent attenuated renal I/R injury (1-4,20). Infusion of extrinsic ATP or ADP was used against brain and ovary I/R (21,22). Normobaric hyperoxia also increases innate antioxidant enzymes against gentamicin induced nephrotoxicity (23).

Although many experimental studies show a decreased injury and preserved renal function after ROS inhibition by antioxidant agents, efficient treatments are still limited (24,25). Currently, the therapy for I/R injury is mainly based on supportive care and fluid administration (24,26).

In summary major renal injuries follow I/R take place in two steps: one, ischemia (hypoxia) that induces ATP depletion and leads to epithelial tubular cells or endothelial cells injuries; and second, reperfusion that leads to oxidative stress, nitrosative stress and inflammation.

With respect to above background may be said that the major trigger of pathogenesis in renal I/R are ATP depletion (Hypoxia), ROS and in follow activation of other pathogenetic pathways. By these insights the new suggestions introduce to investigate in animals and especially clinical trial in combat with renal I/R injuries.

- Compensation of ATP depletion via pretreatment with ATP and AMP injection.
- Inhibition of hypoxia through normobaric or hyperbaric hyperoxia pretreatment.
- Administration of cofilin inhibitor or agents that inhibit actin–cofilin interaction before ischemia.
- Use of powerful antioxidant agents that can increase activation of innate antioxidant enzymes and inhibit complement and inflammation.
- Pretreatment with creatine or creatine phosphate.
- Combination of mentioned suggestions.

## Acknowledgements

The author wants to thank from Bardia Tavafi and Rezvan Bagheri.

## Author’s contribution

Majid Tavafi is the single author of the manuscript.

## Conflict of interests

The author declare no competing interests.

## Ethical considerations

Ethical issues (including plagiarism, misconduct, data fabrication, falsification, double publication or submission, redundancy) have been completely observed by the author.

## Funding/support

None.
